# Diseases of the Windpipe

**Published:** 1838-04-01

**Authors:** 


### DISEASES OF THE WINDPIPE.

1. Observations on the Surgical Pathology of the Larynx and Trachea, including Remarks on Croup, Cynanche Laryngea, Wounds, &c. &c. By *W. H. Porter*, Professor of Surgery in the Royal College of Surgeons in Ireland. 8vo., pp. 275. Second Edition. London, 1837.

2. A Treatise on the Diseases and Injuries of the Larynx and Trachea. By *Frederick Ryland*, Surgeon of the Town Infirmary, Birmingham. 8vo., pp. 328. London, 1837.

3. Traité Pratique de la Phthisie Laryngée, &c. Par MM. *Trousseau* et *Belloc*. 8vo., pp. 488. Paris, 1837. Balliere.

[Concluded from No. LV. page 64.]

In our last Number we entered on the consideration of the diseases of the Larynx and Trachea, and directed the attention of our readers to the general pathology of these parts, and more especially of their mucous membrane. We pointed out some of the peculiar features of inflammation, when it attacks the commencement of the air-passages, and endeavoured to rectify certain errors, which have of late years crept into medical doctrine, in consequence of the adoption of a new term, *Dipthérite*, to designate one form of cynanche or angina. We shewed that instead of regarding *Dipthérite* as a *new*, or hitherto undescribed, disease, it is merely one species of what was formerly described under the name of *Angina membranacea;* and moreover that there are several varieties of this very species, each requiring a peculiar mode of treatment. In the course of our remarks, we exposed the fallacy of some of the most recent French writers, such as Laennec and Bretonneau, in supposing that Dipthérite and Croup are the same disease, differing only in the part affected.

We entered at considerable length into the therapeutic department of the subject, and described what we have found to be the most speedy and effectual means of subduing laryngeal and tracheal inflammation. We then commenced a short account of that distressing malady *phthisis laryngea*, and pointed out some of the most frequent causes which give rise to ulceration of the larynx and its accompanying symptoms. Neglected venereal and mercurial disease is perhaps one of the most common of these causes; and we know that when tubercular degeneration exists in the pulmonary tissue, the mucous structure of the air-passages is not unfrequently affected in a somewhat similar manner at the same time.

In many cases the disease appears to be purely idiopathic, commencing sometimes in the hard, at other times in the soft parts.

At present we propose to call the attention of our readers to *two forms* of laryngeal disease, which hitherto have not been well known to medical men. Decidedly the best description, which we have met with, of these, is that given by Mr. Porter; and we shall therefore avail ourselves duly of his observations to illustrate the subject. These two diseases are *calcareous degeneration*, and *gangrene* or *mortification of the larynx*.

In the former disease, which is comparatively far more common than the other—these cartilages become converted into an earthy, gritty, calcareous matter, which is usually mixed up with portions or spiculæ of carious bone. In the latter, on the other hand, they exhibit the usual appearances of mortified or sloughy cartilage, being "black and dissolved, resembling wetted and rotten leather." Both these forms of morbid degeneration are usually accompanied with the formation of purulent matter around the cartilages. The abscess may burst either outwardly, or into the larynx, and is then rejected by coughing, or into the œsophagus. In the latter case, a communication may be formed between the two passages, and then, says Mr. Porter, the food, but particularly the drink, passing through the ulcer, afford some little insight into the nature of the case.

Let us now consider more minutely each of the two forms of disease.

*Calcareous Degeneration of the Cartilages of the Larynx.*—In this, the laryngeal cartilages become converted into a substance, partly of a calcareous and partly of an imperfectly osseous nature. It is so admirably described by Mr. Porter, that we shall use his own words.

" About the age of 32, and varying from that to 36, we occasionally find that the cartilages of the larynx undergo a remarkable change, and are converted into bone. Previously to and during this process, the structure of these substances is highly organized, and a section of them appears red and very vascular. In most instances the change takes place, like every other operation of the animal economy, without inconvenience; whilst in some particular constitutions a morbid action is set up which terminates in this dangerous disease. It commences usually in the broad posterior portion of the cricoid cartilage, this part being now highly organized, and more capable of producing disease. At first a small earthy deposition is laid down in some part of the cartilage, probably near its centre;—it feels hard and gritty under the knife;—is white as to colour,—and perhaps may be somewhat of the same nature as the earthy degeneration in the coats of arteries. This increases in quantity, so that the entire of the cartilage seems to be converted into it; and as it is totally unorganized, it acts as an extraneous body. An abscess is formed which bursts in one or more places; purulent matter is discharged, frequently mixed with this earthy substance already mentioned, the patient becomes emaciated, and worn down with the cough, difficulty of breathing, and other symptoms that attend this melancholy disease: he either dies with most of the appearances of hectic fever, or, if he recovers, it must be after bronchotomy has been performed, and he breathes artificially for ever after." 131.

From this description, it appears that the morbid action commences in the cartilages themselves. The disease is therefore primarily one of the firm parts. The mucous membrane however of the larynx is necessarily affected from almost its very commencement. It becomes irregular, or corrugated, somewhat thickened, and inflamed. Specks of ulceration soon make their appearance; these spread and coalesce; and thus sores, of the size of a sixpence or shilling, are formed in different parts, while the rest of the mucous membrane becomes more and more congested and disorganized. When, says Mr. P. a larynx is examined, which has long been the seat of this affection, very little trace of its former configuration can be discovered.

Now the morbid changes of the larynx, which we have been describing, may exist for a length of time, without any serious mischief, before any alarm has been excited in the mind, either of the patient, or of his medical attendant.

Slight sore throat, hoarseness of the voice, and a certain degree of uneasiness in swallowing firm morsels of food, are usually the earliest appreciable symptom.

As the mucous membrane of the larynx becomes more decidedly affected, the patient begins to suffer from a most troublesome cough, and from occasional attacks of most distressing breathlessness. These attacks of dyspnœa are, at first, decidedly spasmodic; and sometimes they are so severe, even in the early stages of the disease, that the patient has expired in one of them, even before the existence of the primary malady has been suspected.

Along with the symptoms now mentioned, there is always more or less tenderness of the larynx on pressure.

When the cough has continued for some time, it gradually becomes more moist, the sputa being more abundant and coming away with less difficulty. Enveloped in the sputa, which are now decidedly purulent, are often observed particles—varying in size from a pin's head to a pea—of a gritty, earthy-looking substance. These are small detached portions of the degenerated cartilages.

The breathing gradually becomes more and more distressed, the cough is more incessant, the sputa are more offensive and fetid, and the dyspnœa returns with greater frequency, and severity. These symptoms are usually attended with pain and constriction at some part of the chest, and with all the usual signs of hectic fever; and death inevitably follows.

Having thus described the pathology and symptomatology of this laryngeal disease, we shall shortly allude to its *treatment*,—we do not say its *cure*—before dismissing the subject. No medicine has any effect, as far as we yet know, in arresting the morbid action, when it has once fairly commenced in the cartilages.

Perhaps the establishment of a seton in the side or nape of the neck, and residence in mild and pure country air, with a regulated diet, and extreme quietude of the vocal organs, are all that the prudent physician will think right to recommend. As to teasing his patient with a variety of local and instrumental remedies, he will carefully abstain from such a practice.

Mercury, which is so beneficial in most other laryngeal affections, is worse than useless in this. Certain anti-spasmodics and anodynes may be found necessary to alleviate the cough and paroxysmal dyspnœa; but these will be found of but little avail.

The constitution of the patient is almost always radically and deeply *cachectic*—alas! too often from the previous dissipated and debauched career of his life. Most of the cases of this formidable disease are found to occur in those who have ruined their health by excess either in drinking spirituous liquids, or in venereal pleasures.*

However powerless the aid of medicine may be in arresting the morbid

---

* In the article, in our last Number, we alluded to the frequent occurrence of laryngeal disease in venereal patients. There is manifestly a sympathy, in disease as well as in health, between the organs of the voice and those of generation.

degeneration of the laryngeal cartilages, it is most pleasing to know that, even in the extremity of the evil, the operation of bronchotomy has afforded not only great and immediate relief from suffering, but even a prolongation for months and years, although with the disagreeable inconvenience of wearing a tube in the windpipe for ever afterwards.

This therefore is a point of the greatest importance; and the more so, as the results of Mr. Porter's late experience have most satisfactorily proved to him that the advice which he had given in the first edition of his work—not to perform the operation—is perfectly erroneous. He says that, within the last few years, he has had the gratification of saving the lives of several persons affected with disease of the laryngeal cartilages, by performing bronchotomy; and adds—

"There are at present in Dublin four persons on whom I operated so circumstanced, artificially respiring, without any prospect of being otherwise able to exist for the remainder of their lives. One of these works at the laborious occupation of a stone-cutter, and another is a kind of errand-boy, who subsists by going of messages, and must undergo a considerable deal of fatigue every day. I have, therefore, in now proposing the operation to my patient, very little apprehension of the result, unless there happens to be present some unhappy complication of disease, the most frequent of which is tubercular abscess of the lung." 134.

At first the wearing of the tube is very troublesome and even painful; but gradually the lips of the wound, and the mucous membrane of the trachea become less sensitive, and the patients can remove and replace the instrument without much inconvenience. Mr. Porter mentions that in one of his cases the tube, which had been worn for three years, became so corroded that it broke across in the middle, and a portion of it dropped into the windpipe, requiring a new operation for its extraction. It is necessary therefore for the surgeon to examine occasionally the instrument.

Had our limits permitted, we should have given one or two of the illustrative cases, which Mr. Porter has reported. We must therefore refer those readers, who wish to understand the subject more minutely, to consult the original—which we have already said, is a work of the most practical value.

*The other form of disease of the laryngeal cartilages, which we mentioned, is gangrene or mortification.* This, as might be supposed, is of very rare occurrence. Mr. Porter has witnessed only two instances of it. In one, the affection appeared in an exceedingly acute form, and ran its course with great rapidity; whereas, in the other, it was of a chronic nature, and had been preceded for a length of time by symptoms of laryngeal disease. Both cases occurred about the age of 30. We shall give a condensed report of their most striking phenomena.

*Case* 1. Richard O'Leary was admitted into the Meath Hospital on the 15th July, 1835. His breathing was difficult, laborious, and attended with a hissing noise; the voice was hoarse and ringing; no cough, but severe spasmodic exacerbations every night. On examining the fauces, a large broad and spreading ulcer was perceived on the back of the pharynx.

He had been eight weeks ill, before his admission; and he had taken a quantity of mercurial medicines. The mercurials were continued, and opiates and antispasmodics were administered. He gradually became worse; and on the 25th Mr. Porter was summoned to him in great haste. The dyspnœa was excessive; his voice was gone; and he was evidently in the greatest agony. Mr. P. ordered him a strong antispasmodic opiate; but, this failing to afford any relief, the operation of tracheotomy was performed. For some hours his life was despaired of; but towards evening, after expectorating a large quantity of mucus, he rallied; slept well during the night, and awoke next morning much better in every respect. The discharge through the wound was abominably fetid. For three or four days he seemed to improve; but then his swallowing became more difficult, and the neck was swollen and œdematous. When he attempted to swallow, part of the fluid escaped through the wound.

On the 7th August an incision was made into the side of the neck; the abscess was opened, but no discharge followed. It was found to communicate both with the larynx and pharynx, and was excessively putrid. A large quantity of slough was removed, amongst which was a portion of the right ala of the thyroid cartilage. On the 10th, he died in a paroxysm of convulsion. On dissection, a large abscess existed in front of the larynx and upper part of the trachea, in which the thyroid cartilage lay like a foreign substance, entirely denuded, mortified, and abominably offensive. The front of the cricoid cartilage, and of the two upper rings of the trachea, had been removed by mortification also. The lining membrane of the larynx was thickened, corrugated, and had a granular appearance: part of it was ulcerated, at which point the abscess had communicated with the pharynx.

Case 2. Cath. Young, 30 years of age, applied as an out-patient on the 21st July, 1824, complaining of sore throat, difficulty of deglutition, and pain over the thyroid cartilage. The front of the neck was hard and swollen, but not discoloured. She was slightly feverish. She attributed her illness to the swallowing of a small bone; but this was never found. On the 26th, the tumor broke, and discharged an abominably fetid pus mixed with some mucus. On the 30th, her state was more unfavourable. The pain on swallowing, or cough, was more severe. Her speech was low, and attended with difficulty. There were two ulcers in the front, and these communicated with the trachea. Large loose portions of the thyroid cartilage were seen at the bottom of the sores, of a dark brown colour: the discharge was insufferably fetid. When she attempted to swallow, the fluids occasionally escaped from the wounds. She died on the 1st of August; but a dissection was not permitted. Mr. Porter, however, whilst examining the sores in the dead body, pulled out with a forceps nearly half the left ala of the thyroid cartilage, which was brown, fetid, and putrid; its edges were softened as if by maceration, its centre hard and more resembling horn than cartilage.

We have now completed our description of the morbid degenerations of the laryngeal cartilages. It has been seen that both forms are accompanied with the formation of an abscess around the diseased structures; but that we have regarded this latter process as secondary to, and induced by, the existence of the morbid change in the cartilages themselves. There cannot be a doubt that these structures, or at least their investing perichondrial membrane, are occasionally the primary seat of diseased action; just in the same manner as the bones or their periosteal coverings are known to be. The mischief, in either case, commences in the hard parts; and where this has existed for a certain time, the adjacent soft parts are excited to inflammation and consequent suppuration.

But it is to be remembered that an abscess, and that too of a most unhealthy character, does occasionally form around the laryngeal cartilages, without these being necessarily diseased, at least in the first instance.

In consequence of the strong fascia of the neck, all suppurative swellings here are necessarily confined and pressed inwards, so as to cause, very often, great embarrassment in breathing and even in deglutition. Fluctuation is always very indistinct; and the obscurity of this symptom is usually increased by the œdematous swelling which is present.

Whenever, therefore, there is reason to suspect an abscess in this part, a deep incision should be early made, so as to relieve the tension, and give exit to the matter. Often, indeed, no discharge follows at the time; but, independently of the immediate relief to the difficulty of breathing and deglutition, the matter usually makes its appearance in the course of a day or two. Mr. Porter narrates an interesting case in illustration of this remark; and proceeds to state:—"Since that period I have tried a similar practice in several cases, in some of which I have cut down upon the abscess at once, and in others have left it to open into the wound, and the principle has been acted upon with the most decisive success by Dr. Graves, in cases of abscess of the liver.—The last, and I think the most interesting, case of this nature, under my care, was one in which there was no external trace of the existence of abscess, except a slight fulness on the left side of the lower part of the neck, apparently immediately over the carotid artery. From some circumstances connected with the case it was deemed inadvisable to operate low down; and by an incision in the median line of the neck I merely laid bare the lower part of the larynx, and about the three superior rings of the trachea. Yet this answered every purpose. On the following day a profuse discharge of matter took place from the wound, which pressure on the lower part of the neck shewed to have come from the seat of the suspected abscess; and the introduction of a curved probe, which passed downwards and backwards to the extent of two inches and a half, made it plain that the matter had been situated deeply between the trachea and œsophagus."

The most usual situation of abscess is behind the broad portion of the cricoid cartilage. As a matter of course, any swelling here must soon affect the entrance of the glottis, and often too the passage of the œsophagus. The symptoms however are often most puzzling and unsatisfactory; and the management of such a case will require great nicety, as well as decision, of judgment on the part of the medical attendant. It is not possible to lay down any instructions, which can guide him in all cases. This however we may state that, whenever we suspect that an abscess is compressing the larynx, an outward incision should be made upon it; but that, if the dyspnœa becomes still more alarming, and seems to threaten the life of the patient, recourse must be had to bronchotomy—the subsequent treatment depending on the state of the case.

Having thus discussed some of the most interesting, and least known, idiopathic or spontaneous diseases of the larynx, we now propose to direct our readers' attention to various injuries of this organ; and the first we shall select are the

*Accidents from swallowing strong Acids, boiling Water, &c.*

The caustic poisons are sometimes made use of by the suicide to destroy life, and it is really sometimes astonishing what quantities have been swallowed, not only at once, but also in successive draughts with considerable intervals of time between.

Mr. Porter mentions the case of a young girl who, after taking a quantity of strong sulphuric acid, sat quietly down to tea with some friends, although the dose was so strong as to induce death in a few hours; and also that of a man, who took a second dose of the same acid, because he thought the first might not be sufficient.

In such cases as these, the injury is usually confined to the mouth, œsophagus, and stomach. The larynx escapes, at least generally so; and hence the respiration is very seldom affected. Should, however, symptoms of alarming laryngitis supervene, the surgeon may find it necessary to have recourse to bronchotomy.

The case, however, is very different when the caustic fluid is inadvertently —and not designedly, as with the suicide—swallowed; for no sooner is the person aware of the mistake, whether by the taste or in any other way, than all the muscles of the fauces and throat become spasmodically affected, and the fluid is ejected, partly by the mouth, and partly perhaps by the nares, while perhaps a few drops find their way through the glottis. As a matter of course, all the parts, with which the acid has come in contact, are injured more or less seriously, and are speedily attacked with violent inflammation. The operation of bronchotomy is, as we might expect, more frequently required in this description of case than in the former. All will depend on the violence of the laryngitic symptoms.

The history of the following case will be read with interest. A young girl, being taxed by her mistress with dishonesty, attempted to destroy herself by drinking dilute sulphuric acid. She refused to tell what she had taken; and it was only by observing the effects of some of the fluid, which had been accidentally spilled upon her dress, that its nature was discovered. Very fortunately she had taken a hearty breakfast before committing the act. The rest of the description we give in Mr. Porter's own words:—

"The tongue was white, and its integument peeled off on the second day after the accident, like a piece of thick paper, soaked in wet, and curiously marked by pin-holes of different sizes. The sufferings of this creature were extreme, very protracted in duration, and of a character that I have not seen described. After the violence of the gastric symptoms had somewhat subsided, she was seized with symptoms resembling stricture of the œsophagus; the swallowing of any solid material was perfectly impossible, and the attempt attended with great pain; fluids, if taken cautiously, seemed to stop for some minutes in the œsophagus, and then passed the obstruction slowly; but, if there was any unguarded haste, they were forcibly thrown back through the mouth and nose. At the same time a most profuse discharge of saliva took place; the hospital tray, which held more than two quarts, was filled three or four times a day with this fluid. When these had been removed, symptoms of an hysteric character made their appearance, with which the patient was during a long time teased, and more than seven months elapsed before she could leave the hospital. A very few days after the accident, she exhibited some slight symptoms of laryngeal distress, which were soon removed by the application of a few leeches externally to the throat." 182.

In another case alluded to by the author, which proved fatal in the course of a few hours, the root of the tongue and back of the pharynx were found black and charred; the œsophagus, in spots and patches, was blackened, but apparently not disorganized; the lining membrane of the stomach was completely changed, being quite black, and hanging loose, flocculent, and ragged into the cavity of the organ. The superior portion only of the larynx was inflamed, and slightly œdematous.

The other set of accidents—those, namely, which arise from the swallowing, or rather the attempt to swallow, boiling water—more frequently comes under the notice of the surgeon than that we have been describing. Children are generally the sufferers from this most distressing accident. Little aware of what they are doing, they put their lips to the spout of a boiling kettle or urn, and draw in a mouthful or so. The fluid is probably never fairly swallowed, for the throat must be immediately thrown into the most violent contractions, from the intense pain, to expel the scalding contents. It is possible indeed, that in a few cases a mouthful or so is drawn down into the stomach, during the first alarm and suffering of the child. That this, however, is a rare accident may be inferred from the circumstance of the general absence of any gastric distress afterwards. Sometimes the boiling water is not admitted into the fauces, the child being scalded by the steam alone.

Whatever may be the extent of the injury, the symptoms are usually much alike. As a matter of course, the child is found screaming and writhing with pain; he perhaps cannot utter a word, in consequence of the vesicated state of the tongue; all attemps to swallow are difficult, or quite impossible; and the breathing is always more or less disturbed. In the course of an hour or two, the dyspnœa increases, and becomes more or less croupy; the face is flushed, and the usual symptoms of synochal fever supervene. These, however, are soon exchanged for all the indications of rapid exhaustion; the respiration is more difficult, and is accompanied with a stridulous, piping sound; the face becomes livid, the extremities cold, the pulse weak and faltering, and convulsions perhaps come on. The child dies, either asphyxiated and with struggles, or comatose from exhaustion. The asphyxia is attributable to the closure of the glottis, in consequence of laryngitic inflammation; and the coma from exhaustion, to the very remarkable tendency to atony of the vital powers in all serious cases of burns. When the case is more favorable, the progress to recovery is always very slow and tedious, and is usually much retarded by the proneness which exists to tracheal and bronchitic inflammation, occurring in a state of the system liable to extreme depression.

The pathological appearances are inflammation and vesication of the tongue, cheeks, and fauces; the epiglottis and edges of the laryngeal opening are inflamed and œdematous; and, when life has been prolonged for two or three days, the trachea and bronchial tubes are frequently congested and tumefied, and found to be filled with a large quantity of thin mucus. In a few cases, we meet with a genuine exsudation of coagulable lymph on the surface of the trachea and larynx, analogous to what we find after fatal croup. In other cases, the pulmonary tissue is inflamed and partially hepatised. In reference to the lesions of the œsophagus and stomach, Mr. Porter observes:—

"I have not myself yet seen an instance of the lower part of the œsophagus or of the stomach having sustained injury; a fact which proves how very infrequently the hot water is actually swallowed, and which may possibly be explained by its having been taken accidentally, and without the knowledge or intention of the patient. Neither have I observed stricture of the œsophagus, ptyalism, or any of the other symptoms that are met with in patients who recover after having really swallowed some of the caustic acids." 183.

The *prognosis* in all cases of accidents from swallowing boiling water, or any highly irritating or caustic fluids, is decidedly very unfavourable. It is well known how dangerous a disease laryngitis is in all its forms. When it is induced in the way now alluded to, it is very generally fatal, in consequence of the extreme rapidity of its symptoms, and the accompanying morbid state of the mouth and fauces.

With respect to the *treatment*, it should be vigorously antiphlogistic; as the only chance of safety consists in arresting, or in subduing the laryngitic inflammation. Leeches should be applied freely to the neck and throat, or, if the patient be old enough, general venæsection may be practised.

Mr. Ryland recommends the use of a blister—raised by means of a strong solution of the blistering fly in acetic acid—to the upper part of the sternum at the same time.

Dr. Wallace, of Dublin, has powerfully insisted upon the benefits of administering calomel in large and repeated doses—two or three grains every hour or two, until the system is affected, or a decided impression is made on the disease. In some cases deglutition is quite impossible; and no tube can be passed into the œsophagus, either from the mouth, or from the nostrils. In reference to the operation of tracheotomy, it is instructive to learn the sentiments of so experienced a surgeon, as Mr. Porter. He remarks:

"The fact is, that the operation is always delayed too long; at least, it is longer postponed in this than in other cases of acute laryngeal disease, and until the patient's condition is unfavourable for any operation, but more particularly for such as this one, performed on a person of an age so tender."

"Another source of failure is in the quantity of blood lost by the little patient during the operation. This is certainly true of all cases of bronchotomy performed on the child, where the loss is proportionally much greater than in the adult, and sometimes is so profuse as to embarrass the operator, and even bring the patient's life into imminent peril. Now, it is an observation established by the unerring test of experience, that persons who have suffered from obstructed respiration bear the loss of blood badly, and that, in proportion to the severity of the dyspnœa and the length of time it has endured. In the cases of swallowing boiling water, I have already stated that the difficult breathing is allowed to proceed to the utmost limit possible, and I believe that the loss of blood which, except in some cases of extraordinary good fortune, inevitably occurs in the operation on the child, is by no means an infrequent cause of its want of success." 186.

Mr. Ryland has with praiseworthy diligence collected the details of numerous cases of the accident, which we have been describing. Treating of the operation of tracheotomy he mentions that;—

"Dr. Burgess performed the operation, in one instance, two hours after the occurrence of the accident, and the patient completely recovered. 'The usual treatment for inflammation was pursued' after the operation. Dr. Wallace practised it successfully twelve hours after the accident; the child had previously taken eighteen grains of calomel, and this medicine was continued, in smaller doses, for four days afterwards, indeed, till all obstruction to respiration by the natural passages was removed. Dr. Smyth performed tracheotomy with success twenty-four hours after the accident; in this case also the calomel treatment had been commenced several hours before the operation was undertaken, and was continued afterwards till salivation occurred. Mr. Adams performed the operation successfully nine hours after the accident; bronchitis ensued in the course of a few hours, but the patient recovered by the 14th day." 270.

With this we close our remarks on the injuries of the larynx, which are usually induced by swallowing boiling water or any strong caustic fluid. The next subject for enquiry, in Mr. Porter's work, is the presence of

*Foreign Bodies in the Larynx and Trachea.*

Of late years the attention of surgeons has been more frequently drawn to this accident than formerly, in consequence of the *apparently* greater number of cases occurring now than used to be the case. We say *apparently*, only to intimate that the symptoms of the accident are better known in the present day than in the time of Louis, Pelleton, &c. There can be no doubt that many a child has died from the entrance of some foreign body into the air-tube, while the true cause of the mischief was not ascertained, and the death was attributed to croup or bronchitis. The most prominent symptoms are cough and dyspnœa; and these, in many cases, are not constant, but recurring only at intervals. The young patient is often utterly unaware of what has happened; and hence the surgeon can derive but little information from the tale that is told him. Mr. Porter alludes to the great assistance which auscultation has afforded in the diagnosis of the present accident. It has converted, says he, doubt and indecision into certainty; it has rendered the fact of the presence of a foreign body within the trachea cognizable to the sense; and in imparting to the practitioner that confidence which a correct knowledge of his subject alone can bestow, I am satisfied, it has already contributed to the preservation of numerous lives.

If the foreign substance is loose and moveable up and down in the air-tube, it may frequently be heard distinctly to strike against its parietes with a peculiar râle, or rattling noise: this sound is usually most obvious when the patient makes a forced expiration, and the foreign body is driven up towards the larynx.

On the other hand, if the substance is impacted or fixed at any one point, these auscultatory phenomena are not present; but then those which arise from the partial, or complete at one part, obstruction of the entrance of the air will necessarily be heard. Hence it is, that whenever a foreign body is fixedly lodged either in the windpipe or in one of the bronchi, there is either a feebleness, or a total absence of the respiratory murmur in the *right**

* It was first observed by Mr. Key—and the correctness of the observation has been confirmed by the experiments and researches of others—that a foreign body, when it descends below the trachea, almost invariably occupies the *right*, and not the left bronchus. It would seem from the trials of Mr. Goodman, an able Dublin anatomist, that such is the case even in the dead subject, when any extraneous substance is dropped into the windpipe.

lung, although that side of the chest shall sound as clearly on percussion as ever.

"This occlusion," continues Mr. Porter, "of the lung seldom lasts any continued length of time, and, when the foreign body changes its position, the air gains free admission, and respiration becomes equal in both lungs. When it is continued, that is, when the foreign body is impacted in the bronchus by its size, its weight, or by being entangled in mucus, the patient very often suffers the most intense distress and seems to approach the verge of suffocation; but of this he gradually becomes relieved, as if the system could accommodate itself to the diminished quantity of air. The absence of respiration in the right lung, and its sudden re-establishment, appears to be pathognomonic of the presence of a foreign body; at least I know of no other accident or disease that could occasion such phenomena." 202.

With respect to the rational symptoms indicative of the presence of foreign bodies in the air-passages, they, as may be inferred from the preceding remarks, are any thing but satisfactory. There may be uneasiness or pain at some part of the throat; but this is by no means uniform. In a case, where so jagged a substance as a portion of a large molar tooth was admitted into the windpipe, the patient did not complain of more than a feeling of undefinable uneasiness in the chest, a sensation of weight in breathing, and a tendency to draw heavy sighs, which kept his mind in a continued state of inquietude; yet a more irregularly-shaped substance can scarcely be conceived.

The amount of local distress will depend, in a great measure, on the situation of the offending body. If it be lodged in the larynx, the symptoms will be severe and almost incessant, and may very speedily cause death; but if it is so placed that it cannot materially interfere with the passage of the air, the acute distress soon subsides—only however, in many cases, to be followed by ulceration, and consequent hectic fever; and thus the patient perishes at length with every indication of Phthisis laryngea. Occasionally the body, which had remained impacted for a length of time, becomes accidentally dislodged; and then all the early symptoms of threatened strangulation supervene. Sooner or later almost every case of the accident, of which we are now treating, proves fatal.

But to return to the *semeiology* of such cases, we may remark that there is almost always great restlessness, and anxiety; and, occasionally too, an appearance of emphysema above the clavicles—attributable probably to a rupture of some of the pulmonary vesicles in the upper lobe of the lung. To shew however that we cannot depend upon the merely *rational*—as they have been absurdly called—signs to indicate the existence of any foreign body in the air-pipe, the following case needs only to be mentioned. Mr. Porter was requested to examine the body of a child, who, *it was supposed*, had died from being thrown down by a gig, and from one of the wheels having passed over the chest. She had so far recovered from the immediate effects of the accident that she walked home without assistance, although from the moment that the accident occurred, her breathing had been croupy. She was every now and then seized with fits of most distressing cough and general restlessness. These symptoms continued for 38 hours, when, having arisen for a moment, she expired in a paroxysm of convulsive cough. On dissection, the thorax and its contents were found quite exempt from the slightest injury; but on opening the larynx there was discovered part of an almond-shell, its rough and broken edge entangled in the rima glottidis, and placed in such a manner that it effectually closed up the aperture for the transmission of air. The nature of the case was now made evident. The child had the fragment of shell in her mouth at the time she was struck, and either from the fright or the shock had unconsciously made an effort to swallow, and it passed into the trachea.

Nothing can be more satisfactory than the history of this case: the presence of any foreign body in the windpipe had not been even suspected. Such, in short, is the nature of not a few *supposed* instances of sudden and rapidly fatal croup or suffocative catarrh.

Here we may remark that the accident, now under consideration, almost invariably happens when the patient is suddenly surprised at the time that any small body is in the mouth. The cause of this is obvious. There is an involuntary effort of inspiration made at the moment; and thus whatever is in the current of the inhaled air is drawn into the windpipe at the same time. It is quite a mistake to suppose that the entrance of a foreign body into the larynx takes place during the act of swallowing. Nature has provided too well against the occurrence of this accident; and it is very remarkable that, even when the epiglottis has been partially or almost totally destroyed by disease, it seldom or never takes place. But it is different when a man attempts to draw a full inspiration, whilst any foreign body is within reach of the current of air about to pass into the lungs. At this time the epiglottis is raised, the rima glottidis is distended, and every thing appears to favour the entrance of the air, and of course of whatever it bears along with it. Thus, a person holding a sup of wine in his mouth, to enjoy the flavour, incautiously attempts to breathe: a drop of the fluid enters the larynx—it produces great irritation, and the spasmodic cough that ensues throws it out with great violence, perhaps even through the nostrils. The same accident happens from sucking up an egg, on the top of which some loose salt has been placed: the salt, during the act of inspiration, flies into the larynx, and I have known many persons almost reduced to the verge of death by an occurrence apparently so simple. Thus, in like manner, a bead, a shell, or any thing held incautiously in the mouth, will naturally follow the course of the air; and, in the event of a full inspiration incautiously made, will certainly pass down into the trachea.

So much for the manner in which the accident usually takes place. We have already said how much the violence, and even the character, of the symptoms is influenced by the situation in the air-pipe, which the foreign body may occupy—according as it may be fixed, or loose and moveable; and, if the former be the case, according as it may be impacted in the ventricles of the larynx, or in one of the bronchi.

When the upper portion of the larynx is the seat of the injury, the irritation and other symptoms are always excessive, and the patient may perish in the course of a few days, or even more quickly, either from inflammation, or from spasmodic closure of the glottis. If however the body be fairly lodged in one of the laryngeal ventricles, it has been known to remain there for many years without much inconvenience. The same may be said, when it is fixed low down in the trachea, or in one of the bronchi—the *right* one, as already explained, being almost always the seat. Let it not however be supposed that, under such circumstances, the life of the patient is not endangered. Mr. Porter very justly remarks:

"If the case, however, is allowed to progress without surgical interference, death is certain to ensue, although at different periods after the accident, and under different pathological circumstances, sometimes from imperfect respiration the lung becomes congested and loaded, and the patient dies convulsed on account of the brain being supplied with an improper quality of blood; sometimes life is terminated by bronchitis, pneumonia, or pleuritis; and sometimes, at a still more remore period, death is preceded by symptoms of consumption. In proof of the degree of uncertainty that attends these cases, I need only notice that the subject of Mr. Houston's case died on the 11th day, after having suffered from every form of inflammation to which the parts within the chest are liable; whilst Mr. Liston's patient was operated on six months after a fragment of bone (a substance very analogous to a broken tooth) had passed into the trachea, and recovered in the most satisfactory manner." 204.

Such therefore being the danger of all cases, without exception, in which foreign substances are admitted into the air-tube, there cannot be a doubt as to the propriety of laying it down as a general rule of practice that we should have recourse to the operation of bronchotomy, whenever it is fairly ascertained that the efforts of nature are unequal to the removal of them. As to the use of emetics, with the view of exciting violent expiratory struggles, Fabricius Hildanus objects with great justness to them, on the ground that the accident itself will excite sufficient cough, without the assistance of any therapeutic means. With respect to the proposal of M. Dessault, to introduce an elastic tube from the nares into the windpipe, surgeons are not likely to differ in totally condemning it, as not only useless, but worse than useless. We shall close our remarks on the subject of foreign bodies in the air-tubes, by a brief report of an interesting case related in Mr. Porter's work.

A boy, five years of age, was taken to Mr. Smyly, in consequence of a violent convulsive cough, which had come on quite suddenly, and continued without ceasing. All the information which could be obtained from the little sufferer was, that he had swallowed something, and that it had stuck in his throat. The face was swollen, the eyes were red, and the respiration hurried; but the child spoke without difficulty or pain, and the cough had begun to subside in frequency and force. The auscultatory phenomenon was an intense *puerile respiratory noise* over all the left lung, while over the right scarce any murmur was audible. Both sides of the chest were equally resonant on percussion. The nature of the case being evident—viz., that some foreign body was lodged in the right bronchus—the operation of tracheotomy was performed forthwith. On the trachea being opened, violent fits of coughing took place, and succeeded each other with great rapidity. Several attempts were made with a long forceps to dislodge the foreign body, but without success; the instrument was too thick, and its presence in the trachea caused the most intense distress: a probe was tried, but was not sufficiently long to reach it. These efforts occasioned cough, with great irritation, which obliged us to desist. The patient was then placed in bed, a silver canula having been introduced to keep the wound open. During the night, the breathing became very difficult; and next morning he was apparently moribund, in a comatose state. A long gunshot probe was passed down along the trachea, and fortunately reached and dislodged the offending substance. A violent fit of coughing succeeded, and a kidney-bean was expelled, followed by a large quantity of ropy mucus. Immediately afterwards, the respiratory murmur was loudly audible over the right side of the chest. From this date, in spite of some symptoms of threatened bronchitis, the case advanced to a most favorable termination.

The next subject we select for a few remarks is that of

*Wounds of the Larynx and Trachea.*

No cases in surgery are more distressing when, as is usually the case, the accident has arisen from the act of the suicide. It is a melancholy fact, too, that such cases very frequently prove fatal, even when the injury inflicted is not very serious or extensive. There cannot be a doubt that the state of mental disquietude and gloomy apprehension of the poor sufferer interrupt, so to speak, the usual reparative efforts of nature, and thus have the effect of converting wounds, of by no means very alarming character into unhealthy sores, which, by keeping up a constant irritation of the system, ultimately destroy life.

The influence of the mind on the process of healing is well known to the military surgeon: wounds are much more difficult of cure in the *defeated* than in the *victorious* army. Independently of the unfavorable influence of the mind on the progress of suicidal injuries, their danger is, in many cases, much increased by the length of time which may intervene between the infliction of the injury and the requisition of surgical assistance; but this consideration is of much less importance than the former one.

It is but too true that few suicidal patients ever recover *by compulsion*; and hence it is that the most favorable symptom, in all such cases, is the expression of sorrow on the part of the sufferer for the act of which he has been guilty.

In almost all the attempts of the suicide to destroy life by cutting the throat, the knife passes between the os Hyoides and the upper edge of the Thyroid cartilage; and, from the head being thrown back at the time and the throat stretched to its utmost, it enters, not the larynx, but only the cavity of the mouth. In some cases the epiglottis escapes all injury: in others, however, it is more or less severed. This circumstance is of considerable importance in determining our prognosis of the event.

By attending to the direction of the knife, as now mentioned, we can at once understand how it is that the large blood-vessels of the neck *generally* escape: the anatomist knows that on the parallel of the thyro-hyoid space they lie deep in, as it were, a hollow. In short, it is certainly not so much from the importance of the parts divided, as from collateral and co-existing circumstances, that suicidal wounds of the throat prove so generally fatal in the long run. How often is it that the unhappy sufferer remains permanently gloomy and sullen, expressing neither regret for his crime, nor any satisfaction at the failure of his dreadful attempt! Perhaps too he will refuse to take any sustenance, and will resist the surgeon's wish to introduce food into the stomach by means of an œsophageal tube. Mr. Porter alludes to a case of this description, where the patient would receive no nourishment, except what could be derived from enemata of broth and milk. He survived for several months, and during this time the wound, although frequently closed by suture and agglutinative plaster, could not be made to unite. This man, as is usual, suffered from attacks of bronchitis; and an enormous quantity of mucus was secreted, all of which was expelled through the wound, no matter what pains were taken to prevent it. At length he perished, a perfect example of death from inanition, and one of the most melancholy spectacles that humanity could exhibit.

The difficulty of maintaining the head and neck motionless, and the constant passing of the food, saliva, and mucus through the lips of the wound, are doubtless the chief causes which prevent the agglutination of the divided surfaces. Then too, if any cough be present—and how frequently is this the case—every succussion of the chest and throat must necessarily disturb the wound.

It thus appears that, even under the most favorable circumstances, the management of wounds of the neck, penetrating either into the cavity of the mouth or into the air-passages, is attended with much difficulty, and requires the greatest skill on the part of the surgeon. Hitherto it has been too much the custom to follow one uniform line of treatment in all cases—viz., to stitch the gaping wound immediately, and plaster it up with adhesive strips. We are satisfied in our minds that this indiscriminate practice has often been very pernicious. The irritation of the sutures, increased by every movement of the neck, and the plugging up of the wound with saliva and mucus, have, in too many instances, aggravated the very evil which the surgeon was so anxious to rectify. Hence he is obliged, in the course of a few days, to remove the stitches; and, instead of finding the wound at all disposed to cicatrise, he discovers it looking any thing but healthy, and gaping wider than ever.

It is a much more judicious plan to leave the wound easy and undisturbed for two or three days, the surgeon being satisfied with using all means to keep the head and neck in an appropriate position, so as to approximate the lips of the wound, and administering all food by means either of an œsophageal tube, or of enemata *per rectum*. If indeed the wound be very extensive, a stitch or two at each side may be employed; but as to sewing it up with several sutures the practice is, in our opinion, positively injurious—at least in very numerous instances.

When the irritation of the system, and perhaps too the disquietude of the mind are subdued—provided the wound of the neck look favorable and disposed to assume a healthy healing action—the surgeon may, with much better prospect of success, endeavour to draw together and to retain the divided edges.

English surgeons have, we think, too indiscriminately, not only in the present, but in other classes of wounds, aimed at the healing *by the first intention*. In many cases of amputation it will be found a wise and a good practice to use the gentlest means only to retain the divided edges in contact, for the first few days after operating. The proper position of the limb, and the use of a few turns of a well-applied bandage, are all that is sufficient for the purpose. How often have we seen in British hospitals the wound of the stump, on its first dressing, looking puffed, swollen, and angry, adhering imperfectly only at one or two points, and oozing out an unhealthy sanious discharge.

All this was the effect of the *forcible* drawing together of the divided surfaces by means of sutures, adhesive plaster, and rather tight bandages. True it is that these evils might have arisen, in part at least, from the flaps of the wound having not been made sufficiently long to meet without these approximating means; but, even allowing that such was really the case in some instances, we are satisfied that the practice alluded to is often most unwise and hurtful, as an invariable and indiscriminate mode of treatment.

The same remarks apply to many wounds of the scalp, and indeed of other parts of the body. We have often seen a wound, after the removal of a diseased mamma and of other tumors, heal most kindly, when neither sutures nor adhesive plaster had been applied, and a mere pledget of lint dipped in water had been laid over it, and the divided edges supported, and in some degree approximated, by a well-applied bandage.

No one can dispute the pre eminent advantage of *primary* over *secondary* union in cases of wounds of the throat. The extreme difficulty, amounting in but too many instances to an absolute impossibility, of effecting an adhesion *by the first intention* is however a great obstacle to the best-directed efforts of the surgeon; and therefore, instead of *invariably* resorting to the closure of the wound *at once*, we feel confident that the plan, which we have recommended above, would be found in general practice to be more successful.

It may be almost unnecessary again to urge the extreme importance of the most perfect quietude not only of the injured parts themselves, but also of the whole system. For the latter purpose, it will be often advisable to draw blood from the arm, if there has not been considerable hæmorrhage from the wound. The phrenzied excitement of the suicide will be best allayed by this practice.

To ensure as complete a repose of the injured parts, it is quite necessary to prevent all attempts to swallow food; and hence the necessary nourishment should be administered either by the *œsophagus* or by the *rectum*.*

In passing a tube into the stomach—and this should be always done, if possible, from the nares, and not from the mouth—caution is necessary that it does not enter the larynx instead of the œsophagus.

"Any elastic substance," says Mr. Porter, "introduced by the nostril will strike the spine nearly behind the uvula, and its point will thus be directed forwards and downwards, instead of backwards and downwards, so that its natural tendency will be to pass into the larynx, and not into the œsophagus. Nor will a lighted candle held before the orifice of the tube, which is the best criterion we have to judge by, prove a certain test, unless it be persevered in for a given time, so as to shew the regular alternations of inspiration and expiration. It is very easy to conceive, that air may pass through a tube from the stomach, particularly at its first introduction; and on the other hand the instrument might be in the trachea, and yet no air pass through it, in consequence of its being

---

* Mr. Porter seems to give a decided preference to the latter mode of supporting the patient's strength. Alluding to a suggestion of his own to perform bronchotomy in certain cases, he says:—"For the rest, I would not permit an attempt to swallow even a drop of liquid, because I know the patient can be sufficiently nourished without it: I would not try to introduce a tube into the œsophagus, because the effort produces great irritation and violent paroxysms of cough." We are much inclined to take the same view of the question.

choaked with mucus, or lying entangled in a fold of the lining membrane. The cough excited on its introduction will be no criterion; for it is impossible to pass the instrument without more or less irritating the larynx, and thereby exciting its sensibility." 253.

In all cases where it is probable that the œsophageal tube is necessary, the sooner it is introduced, and the less frequently that it is disturbed, the better; as every successive introduction adds for the time to the distress of the patient, and must disturb the healing process in the wound itself.

Hitherto our remarks have been confined to the appropriate treatment of wounds of the throat, in their *earlier* stages only; we shall therefore now allude to some of the difficulties attending the management of these accidents at a *later* period of their progress. Not to mention the gradual exhaustion and decay of the strength from a constant irritation, as well as from an imperfect supply of nourishment and other causes, there is apt to supervene, in not a few instances, a subacute inflammatory action and disorganization of the parts around the entrance of the larynx, inducing a gradual contraction of its orifice, and giving rise to a series of symptoms at once most painful and alarming. The constant cough, and the repeated paroxysms of severe dyspnœa, must wear out the strength of the strongest constitution. The patient may perish either from rapid suffocation, or from an irritating hectic sort of fever, or, lastly, from a chronic laryngitis and bronchitis.

Any one of these circumstances will necessarily add greatly to the unfavourable character of the prognosis; and it will be the duty of the medical attendant to devise, if possible, some means to obviate the danger—a danger arising, in most cases at least, from the imperfect supply of atmospheric air to the lungs.

There are two ways in which this may be done—either by passing an elastic tube into the larynx, or by performing the operation of tracheotomy. The former plan was adopted and recommended by the celebrated French surgeon Dessault; but is very ably, and, in our opinion, most satisfactorily reprobated by Mr. Porter. The necessary irritation consequent on the introduction of any instrument into the windpipe—an organ at all times so sensitive to the presence of any foreign body, and the more so now in consequence of inflammation, not to mention its contraction from previous disease—is a most serious objection to its adoption. We have only to recollect, says our author, that the passing of a tube into the larynx is an operation in itself by no means free from danger, that it is extremely difficult to be performed, that awkward and repeated attempts produce inconceivable distress, and that, even when performed with the greatest dexterity, it must invariably excite cough and restlessness—symptoms always disagreeable and often disastrous, particularly where it is desirable to maintain the edges of the wound in contact, or where arteries have been secured by ligature—to hesitate before we have recourse to the practice now alluded to. The operation of tracheotomy is exempt from all these objections, and, although under certain circumstances it may be difficult and even attended with some degree of danger, it is altogether preferable to that of introducing and retaining any sort of instrument in so irritable an organ as the larynx.

It is unnecessary to pursue this subject. We shall therefore proceed in our analytic labours, and, as there are some very valuable remarks in Mr. Porter's chapters on

*Bronchotomy as applicable to the Treatment of Asphyxia,*

We shall select these, with which to close this review.

Our author most truly observes that, in the whole range of medical experience, there is not an accident which demands more practical skill, more patience and assiduity, and assuredly none the successful treatment of which will more amply reward the surgeon, than that of suspended animation.

He has no time to consult books; often too he has not the benefit of consultation with another; all must depend upon himself.

A minute or two may decide his patient's fate, and the happiness or grief of a numerous family. We need not say more to urge upon all to have their minds completely prepared on this momentous subject of professional practice. Mr. Porter's advice is excellent:—

"For a contingency of this description, a man must be always prepared; he must have his resources ready to be brought into operation on the most sudden emergency; for these accidents are almost constantly attended with hurry and confusion, in which, if the surgeon participates in the smallest degree, his patient will probably be lost. Calmness and self-possession are absolutely requisite; and the only means by which they can be acquired, is by studying and becoming familiar with the different forms of asphyxia, the causes that produce them, and the possible complications, both favourable and otherwise, that may be present in each: thence may the proper line of treatment be deduced, and principles established that can be applied without hesitation or delay." 221.

Asphyxia may be induced in various ways. A morsel of food, in the hurry of deglutition, may stick in the *rima glottidis*, and cause very speedy suffocation, partly by mechanical obstruction, and partly by exciting spasmodic closure of the orifice.

"A woman passing along the street, and eating a piece of cake, suddenly fell, gave two or three convulsive struggles, and to all appearance died. She was taken up, and surgical aid almost instantly obtained; the fauces were examined without any appearance of an extraneous body; an elastic switch was passed down into the œsophagus, and as far as the stomach, without meeting with any impediment. Bronchotomy was proposed, but, in consequence of some objection being raised, it was not performed, and the patient was lost. On dissection it was found that this woman had a deficiency in the palate, which was stuffed with rags of lint: these had gotten loose, and became entangled in the morsel she was about to swallow, which was stopped immediately over the epiglottis, and thus kept it closely shut down." 224.

Had bronchotomy been performed at once, this patient would, in all probability, have been saved. Such cases may be readily mistaken for examples of apoplexy, disease of the heart, &c. The following short history will illustrate this remark.

A sailor had been eating in company with another man in an upper room in a public-house: both walked to the stairs, down which he (the sailor) *fell* or *was thrown*, and was taken up at the bottom of the flight quite dead. He was a short stout man, with a remarkably short neck; the face was swollen and very red; the eye-balls staring; and every external appearance denoted that he had died from apoplexy, particularly as no mark of injury could be detected. Mr. Porter was desired by the proper authorities to examine the body, as some suspicion of foul treatment had fallen on the other man. On examining the mouth, he found some potatoes and meat still lying on the tongue and adhering to the teeth; and, on dissecting the parts, he discovered a large piece of half-chewed mutton lying exactly on the epiglottis and effectually shutting it down.

What is the treatment which the surgeon should adopt in such a case? Clearly the object is to restore the passage of the atmospheric air to the lungs. This may be done *either* by removing the obstructing body with the finger or forceps, and then introducing a tube from the mouth or nares to inflate the lungs, *or* by at once performing the operation of bronchotomy and inflating the lungs from the artificial opening.

Mr. Porter gives the preference to the latter method, on the ground chiefly, that less time is lost, and that the most potent resuscitative means—the insufflation of the air—can be more immediately adopted.

The surgeon, however, will act rightly first of all to insert his finger into the mouth as far back as he can, and remove any obstructing body that may be present there: for it is quite possible that the mere irritation of the throat, thus stimulated, may induce the *excito-motory* action of the larynx, and re-establish the movements of respiration. Be this as it may, no one will dispute the excellence of Mr. Porter's urgent advice to lose not a moment of time in any unnecessary operations. We think that he is quite right in preferring an artificial opening of the windpipe to the introduction of a tube from the mouth or nares into the larynx. Independently of the more speedy execution of the former operation, he remarks:—

"There is another circumstance connected with this subject which should decide the surgeon in favour of bronchotomy. It is well known that the powers of life in any patient that has been apparently suffocated are extremely reduced, and that after his restoration it frequently requires the utmost care to prevent his relapsing into his former state again. Thus, it may happen that the process of inflation of the lungs shall have to be resumed five or six times, or even oftener, and this during a very short space of time. If such necessity should be found to exist, there are, probably, few practitioners who would prefer the introduction of a tube through the nostril every time respiration became imperfect; and as for leaving the tube, once introduced, within the trachea, producing irritation and exciting cough, it would scarcely be feasible, and certainly injudicious. On the other hand, the operation of bronchotomy presents the easiest means of inflating the lungs at any moment, and although the necessity of resorting to this procedure may possibly not arise, yet the operator should always bear in mind that in all probability it will, and prepare in the commencement for those contingencies which may subsequently create no inconsiderable embarrassment, or perhaps render all his exertions unavailing." 228.

The remarks which we have made on the asphyxia, which is caused by the *rima glottidis* being plugged up with a morsel of food, are to a certain extent applicable to the other forms of suspended animation. The leading object in all cases is to introduce air into the pulmonary tissue, with the view of re-establishing respiration. All other therapeutic means are secondary, and subsidiary to this; and the only question that is to be considered is, how and in which manner the lungs are to be inflated.

The ordinary method of the surgeon applying his mouth and breathing into the mouth or one of the nostrils of the patient, while the larynx is gently pulled down and pressed back, will succeed in some cases; but it often will fail entirely. The air may, in truth, not be admitted at all into the windpipe; and, instead of the lungs, the gullet and stomach may be distended. It is a mistake to suppose that in all cases of asphyxia—or even when actual death has taken place—that the *rima glottidis* is quite pervious. In not a few instances has the epiglottis been found on dissection to be closely applied to the opening of the larynx, so that all attempts to inflate the lungs, by blowing air from the mouth or nostrils, must be entirely ineffectual. This state has been observed to be, on the whole, more frequently the case in that kind of asphyxia induced by the inhalation of carbonic acid, than in any other. It may be well therefore, whenever we have reason to suspect that the insufflated air does not readily enter the air-tubes, that we either introduce an elastic tube, a catheter or such-like instrument, into the larynx from the mouth or nares, *or* proceed at once to the operation of laryngotomy. The mere division of the integuments and making an opening into the larynx are attended with but trifling danger; and the time that may be thus saved and the direct and easy introduction of the air into the lungs, thus performed, are most important advantages. It is more than probable that, if the operation were more frequently performed at once, and no time were lost in making attempts to inflate the lungs in the ordinary manner, not a few lives might be saved. When we have every necessary and convenient instruments, such as tubes, bellows, &c. at hand, the operation may be generally dispensed with; but summoned unexpectedly, as the surgeon usually is to a case of suspended animation, without the benefit either of assistants or even of the appropriate instruments, we should advise him to open the larynx at the thyro-cricoid space, and, either directly with his mouth or with the aid of a short tube inserted into the wound, keep up a continued and regular insufflation of the chest for a considerable length of time, until natural respiration is re-established.

